# Chloroplast Genome Evolution and Species Identification of *Styrax* (Styracaceae)

**DOI:** 10.1155/2022/5364094

**Published:** 2022-02-24

**Authors:** Yun Song, Wenjun Zhao, Jin Xu, MingFu Li, Yongjiang Zhang

**Affiliations:** ^1^CAIQ Center for Biosafety, Sanya 572025, China; ^2^Institute of Plant Inspection and Quarantine, Chinese Academy of Inspection and Quarantine (CAIQ), Beijing 100176, China

## Abstract

The genus *Styrax* L. consists of approximately 130 species distributed in the Americas, eastern Asia, and the Mediterranean region. The phylogeny and evolutionary history of this genus are not clear. Knowledge of the phylogenetic relationships and the method for species identification will be critical for the evolution of this genus. In this study, we sequenced the chloroplast genome of 17 *Styrax* samples and added 17 additional chloroplast genome sequences from GenBank. The data were used to investigate chloroplast genome evolution, infer phylogenetic relationships, and access the species identification rate within *Styrax*. The *Styrax* chloroplast genome contains typical quadripartite structures, ranging from 157,641 bp to 159,333 bp. The chloroplast genome contains 114 unique genes. The *P* distance among the *Styrax* species ranged from 0.0003 to 0.00611. Seventeen small inversions and SSR sites were discovered in the *Styrax* chloroplast genome. By comparing with the chloroplast genome sequences, six mutation hotspots were identified, and the markers *ycf1b* and *trnT-trnL* were identified as the best *Styrax*-specific DNA barcodes. The specific barcodes and superbarcode exhibited higher discriminatory power than universal barcodes. Chloroplast phylogenomic results improved the resolution of the phylogenetic relationships of *Styrax* compared to previous analyses.

## 1. Introduction

Chloroplasts are involved in photosynthesis and energy transformation in plants [1, 2]. Its own genome is known as the chloroplast genome, plastid gene, or plastome, which commonly occurs in multiple copies within the organelle. The important role of the chloroplast genome is functioning of the photosynthesis and other metabolic processes. The chloroplast genome is 120-160 kb in length [2, 3] and has a highly conserved quadripartite circular organization. This organization contains two single-copy regions (LSC and SSC) separated by two copies of inverted repeat (IR) regions [4]. The chloroplast genome encodes approximately 80 protein-coding genes, four rRNAs, and 30 tRNA genes [1, 2].

Advances in DNA sequencing technology have provided scientists with high efficiency and low cost to obtain complete chloroplast genome sequences. The chloroplast genomes are mostly inherited uniparentally, lack recombination, have compact size; thus, they effectively expand genetic information. Although the genome structure is conserved, mutational events, including indels, SSRs, and single-nucleotide substitutions (SNPs), are frequently occurring even in related species [5, 6]. These mutational resources provide rich information to infer evolutionary patterns [7], establish relationships among the plants [8–10], and provide effective genetic markers to resolve complex evolutionary histories [11, 12]. Moreover, plant DNA barcodes rely heavily on chloroplast genome sequences. Chloroplast genome markers, including *rbcL*, *matK*, and *trnH-psbA*, have been used as core DNA barcodes for plants [13]. Comparison of complete chloroplast genome sequences also provides an opportunity to identify specific plant DNA barcodes [14, 15]. Whole chloroplast genome sequences have been used as superbarcodes for plants in recent years [16, 17].

The genus *Styrax* L. consists of approximately 130 species distributed in the Americas, eastern Asia, and the Mediterranean region [18]. The *Styrax* species have important medicinal, ornamental, and economic values. The seed oil or resin of several species is a valuable medicinal ingredient and raw material for the manufacture of aromatic oils.

The most taxonomic treatment of *Styrax* is from Fritsch [18] who conducted a phylogenetic analysis based on 34 morphological characters. In this treatment, Styrax was divided into section *Valvatae* (predominantly tropical evergreen species) and section *Styrax* (north-temperate deciduous species). The section *Styrax* was divided into two series: *Cyrta* and *Styrax*. Series *Cyrta* were distributed in eastern Asia and eastern North America with serrated leaf margins and included 31 species. Series *Styrax* is distributed in western North America and western Eurasia with entire leaf margins and included three species. The *Valvatae* section also included two series. Series *Valvatae* was a strictly neotropical clade including about 77 species, and the series *Benzoin* was a strictly paleotropical clade including nine species.

The taxonomy of *Styrax* species remains incomplete, and several new species have been published based on their morphological characteristics [19–22]. Several studies have used molecular data to infer the phylogeny of *Styrax* species, such as the nuclear ribosomal DNA ITS [23] and chloroplast markers *ndhF-rpl32-trnL*, *trnK*, *trnL-trnF*, *trnS-trnG*, *trnV-ndhC*, *rpoC1*, and *rpoC2* [23–25]. However, these results showed that those markers had low divergence. Therefore, sampling more genetic characters, such as the chloroplast genome sequences, may enhance the species identification.

To better understand the chloroplast genome evolution in *Styrax* and identify the variable markers to species identification within Styrax, we sequenced the chloroplast genome of 17 samples of *Styrax* and added published data from GenBank. Specifically, we attempted to (1) elucidate the chloroplast genome evolution and (2) determine whether the variable chloroplast markers or whole chloroplast genome data can be effective for *Styrax* species identification.

## 2. Materials and Methods

### 2.1. Plant Materials and DNA Extraction

A total of 17 species of the genus *Styrax* were obtained from the field and the DNA Bank of China, Institute of Botany, Chinese Academy of Sciences, and the DNA Bank of China has been permitted obtaining from the materials of the specimens in PE (Institute of Botany, Chinese Academy of Sciences). The details of the 17 species are shown in Table S1. Fresh leaf tissues from each accession were immediately dried with a silica gel before DNA extraction. Total DNA was extracted using a modified CTAB DNA extraction protocol (mCTAB) [26]. In addition to the newly collected material for DNA sequencing, publicly available complete chloroplast genome sequences (17 accessions, Table S1) of *Styrax* were also included. In total, the dataset of sequenced samples and GenBank accessions consisted of 34 individuals representing 29 *Styrax* species.

### 2.2. Chloroplast Genome Sequencing, Assembly, and Annotation

Chloroplast genome sequencing was performed at Novogene (Beijing) using the Illumina HiSeq X-ten platform. Total DNA was sheared to 350 bp fragments using an ultrasonicator. A rapid library was prepared using the NEBNext® Ultra™ DNA Library Prep Kit. Each sample yielded approximately 4 GB of data.

Illumina data were filtered using Trimmomatic v0.36 [27] to remove the adaptors and low-quality reads with *Q* − value ≤ 20. The parameters were set as follows: leading: 20; trailing: 20; sliding window: 4 : 15; MIN LEN: 36; and AVG QUAL: 20. The clean data were used to assemble the chloroplast genome using GetOrganelle [28], and the *k*-mer length was set to 85, 95, and 105. Complete chloroplast genomes were annotated using Plann [29], and the published chloroplast genome sequences of *S. obassis* (GenBank Accession number: MN560143) was used as the reference. Circular chloroplast genome maps were visualized using OGDRAW [30]. The final annotated chloroplast genomes were deposited in GenBank under accession numbers MZ285733 to MZ285749.

### 2.3. Repeat Analysis and Whole Genome Comparison

SSRs in the chloroplast genome were identified using the Perl script microsatellite identification (MISA) software. The parameters implemented in MISA are as follows: repeat units ≥ 10 for mononucleotides, repeat units ≥ 5 for dinucleotides, repeat units ≥ 4 for trinucleotides, and repeat units ≥ 3 for tetranucleotides, pentanucleotides, and hexanucleotides.

Small inversions were identified based on the aligned chloroplast genome sequence matrix, according to Dong et al. [7]. Inversions form a stem-loop structure, including inversion sequences and inverted repeats at the opposite flanking end [7].

The mVISTA program was used to analyze the variation in the *Styrax* chloroplast genomes [31], for which sequence annotation of *S. agrestis* was used as the reference. The 47 *Styrax* chloroplast genomes were aligned using MAFFT v7.0 and then adjusted manually using Se-Al v2.0 [32]. To explore the sequence divergence with the whole chloroplast genome in the 27 *Styrax* species, genetic *P* distances were calculated with MEGA X [33].

### 2.4. Mutation Hotspots Identified and DNA Barcoding Analysis

Three factors, including nucleotide diversity (*π*), mean distance (*D*), and the proportion of zero pairwise genetic distances (*Z*) for each species in the matrix, were used to explore the mutation hotspots in the *Styrax* chloroplast genome. Nucleotide diversity was calculated using the software DnaSP v6 [34]. Mean window distance and the proportion of zero pairwise genetic distances for each species in the matrix were calculated using the *slideAnalyses* function of the SPIDER package [35] in R. The window length was set to 600 bp, with a 50 bp step size.

Nucleotide diversity and variable and parsimony-informative sites were used to evaluate marker variability. The three universal chloroplast DNA barcodes, *rbcL*, *matK*, and *trnH-psbA*, were also used in this analysis. Nucleotide diversity was determined using the DnaSP v6 software [34], and variable and parsimony-informative sites were calculated using MEGA v7 software [36].

Distance and tree-building methods were used to assess the marker discriminatory power. The distance method uses the *nearneighbor* function of SPIDER. The tree-based method was applied using ML. ML analysis was conducted using RAxML nonparametric bootstrapping and 1000 ML pseudoreplicates. The best-fitting models were selected using ModelFinder [37].

### 2.5. Phylogenetic Analyses

Phylogenetic analysis was conducted to elucidate the interspecific phylogenetic relationships within *Styrax*. Two datasets were created to infer the *Styrax* phylogeny. The first data were whole chloroplast genome sequences of 34 *Styrax* samples with *Huodendron tibeticum* and *H. biaristatum* used as the outgroup. The second dataset was the concatenation of the 80 coding genes. Maximum likelihood (ML) and Bayesian inference (BI) methods were used to infer phylogenetic relationships. All phylogenetic analyses used the best-fitting models of nucleotide substitution selected in ModelFinder [37] under the Bayesian information criterion. Maximum likelihood (ML) analyses were performed in RAxML-NG [38] with 500 bootstrap replicates. The BI tree was inferred to be MrBayes v3.2 [39]. The BI analysis was run with two independent chains and prior for 20 million generations, with sampling every 1000 generations. The initial 25% of the sampled trees were discarded as burn-ins. Stationarity was considered to have been reached when the average standard deviation of the split frequencies remained below 0.01.

## 3. Results

### 3.1. Structural Characteristics of the *Styrax* Chloroplast Genome

Illumina paired-end sequencing produced between 11,971,102 (*S. japonicus*) and 40,957,798 (*S. rugosus*) paired-end clean reads per samples. After screening these paired-end reads through mapping with *Styrax* chloroplast genome using Geneious V9, 67,059 to 1,702,907 chloroplast genome reads were extracted with 64 × (*S*.*americanus*) to 1,618 × (*S*.*roseus*) coverage (Table S2).

**Figure 1:**
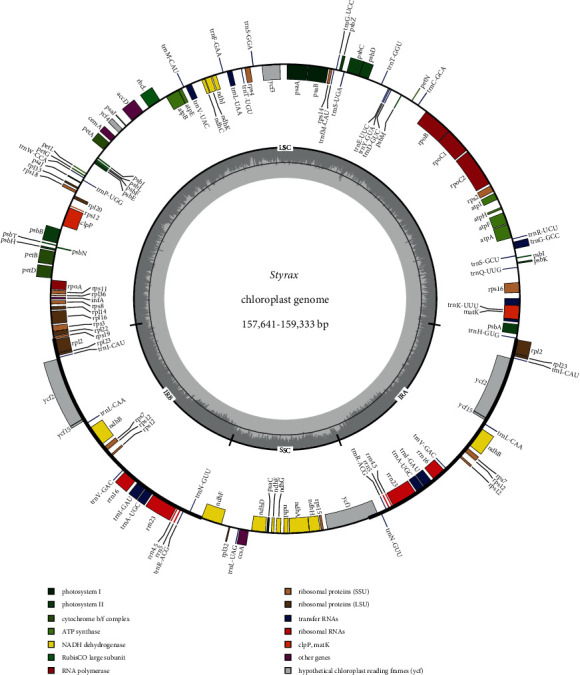
Chloroplast genome map of *Styrax*. Genes shown inside a circle are transcribed counterclockwise; genes outside are transcribed clockwise. Difference functional groups of genes are shown in different colors.

All 17 newly sequenced chloroplast genomes were assembled entirely, and their sequence lengths and structures were very similar (Table 1, Figure 1). The chloroplast genome length ranged from 157,641 bp (*S. japonicus*) to 159,333 bp (*S. suberifolius*). The chloroplast genome has a quadripartite structure typical of angiosperms composed of an LSC region (87,250–88,656 bp), SSC region (17,993–18,412 bp), and two IR copies (26,017–26,352 bp). The overall G/C content was approximately 37%. The *Styrax* chloroplast genome encodes 114 genes, including 80 protein-coding genes, 30 transfer RNA (tRNA) genes, and four ribosomal RNA (rRNA) genes. The mVISTA results revealed collineation, no rearrangement, and high sequence similarity across the *Styrax* chloroplast genomes (Figure S1).

### 3.2. Repeats and Small Inversions

**Figure 2:**
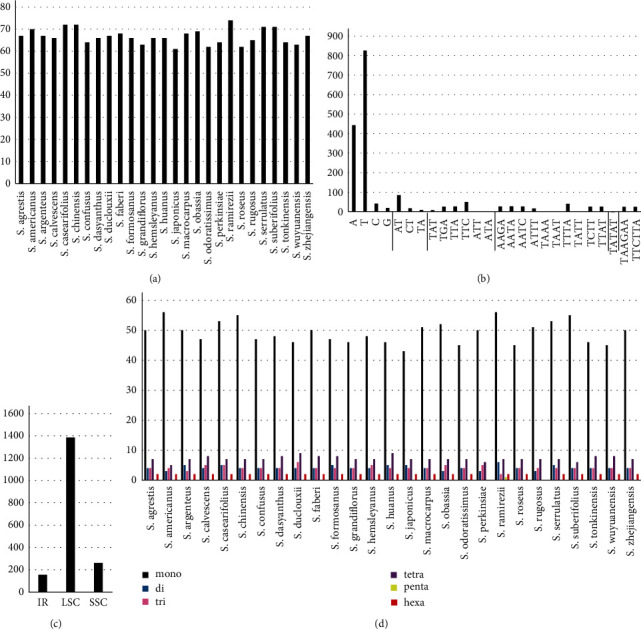
Frequency of SSRs in the *Styrax* chloroplast genomes: (a) the number of SSRs detected in the different *Styrax* species; (b) the number of SSR motifs in different repeat class types; (c) the frequency of SSRs in LSC, IR, and SSC regions; (d) the number of SSR types in different *Styrax* species.

A total of 61–74 SSRs were found in the *Styrax* chloroplast genomes. Mono-, di-, tri-, tetra-, penta-, and hexanucleotide SSRs were identified (Figure 2). The majority of SSRs were mononucleotide repeats in all *Styrax* species, followed by trinucleotide repeats. Pentanucleotide repeats were limited to one occurrence in *S. ramirezii*. Most mononucleotide repeats were composed of A/T with minimal G/C. The LSC region contained the most significant SSRs (76.91%), with 14.49% identified in the SSC region and 8.61% in the IR region.

Seventeen small inversions were identified in the *Styrax* chloroplast genome (Table 2). All inversions and their inverted repeating flanking sequences formed stem-loop structures. The inversion length was 4 to 164 bp, and the flanking repeats ranged from 6 bp to 28 bp. The longest inversion occurred in the *trnS^UGA^* –*psbZ* region. Except for the two inversions, the others were all located in the LSC region. All inversions were located in noncoding regions, including 14 in space and three in intron regions. The *trnF^GAA^ –ndhJ* region included three inversions, and *ycf3–trnS^GGA^* had two inversions. Seven inversions (*trnC^GCA^–petN*, *ycf3–trnS^GGA^* 01, *trnT^UGU^–trnL^UAA^*, *trnF^GAA^–ndhJ* 01, *trnF^GAA^–ndhJ* 02, *clpP*, and *trnR^ACG^–trnN^GUU^*) were specific to one species (Table S3). For example, the inversion in *trnC^GCA^–petN* was specific to *S. duclouxii.* The inversion in *trnS^GCU^–trnG^GCC^* and *psaJ–rpl33* occurred in only one sample of *S. agrestis*, whereas inversions in *trnS^GCU^–trnG^GCC^* and *petN–psbM* occurred in some samples of *S. tonkinensis*. This suggests that these three inversions are polymorphic in one species.

### 3.3. Universal DNA Barcodes of *Styrax*

Three universal candidate DNA barcodes, *rbcL*, *matK*, and *trnH-psbA*, were analyzed to test the species discrimination power of *Styrax* (Table 3). The core barcode of *rbcL* had an aligned length of 695 bp, with 14 variable sites and nine parsimony-informative sites. Nucleotide diversity was 0.00297. The *matK* barcode was more variable than *rbcL*, with an aligned length of 878 bp and 25 variable sites in *Styrax*. The *trnH-psbA* barcode is an intergenic space region with an aligned length of 486 bp. It contained 25 variable sites and 14 parsimony-informative sites. According to the nucleotide diversity values, *trnH-psbA* was the most variable marker among the three chloroplast universal markers.

**Figure 3:**
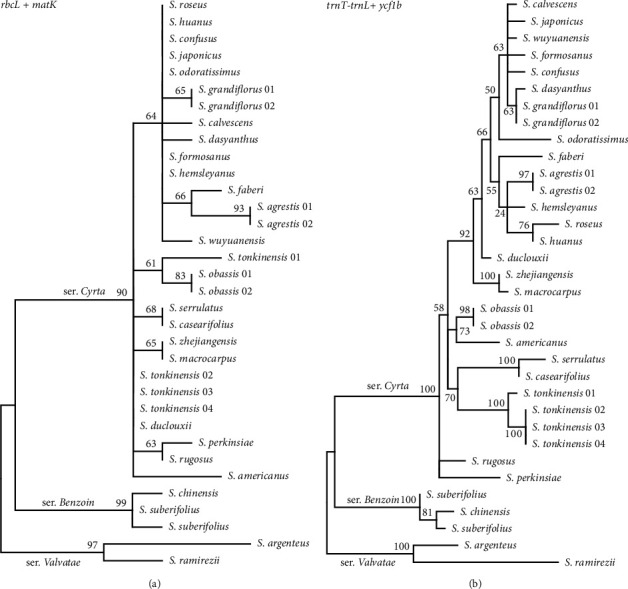
Phylogenetic tree of *Styrax* based on gene markers: (a) standard DNA barcodes (*rbcL+matK*); (b) specific DNA barcodes (*trnT-trnL+ycf1b*).

Using the distance-based species identification methods, the three universal DNA barcodes had 18.4% (*rbcL*), 44.4% (*matK*), and 48.8% (*trnH-psbA*) discriminatory power in *Styrax*. Combining *rbcL* and *matK*, the success rate was 48.15%, and the success rate of the combined three barcodes was 70.37%. The results obtained using the tree-based method are shown in Figure 3(a). The phylogenetic tree had a lower resolution and lower support values. Four individuals of *S. tonkinensis* did not form a monophyletic clade.

### 3.4. Identification of Specific DNA Barcodes of *Styrax*

**Figure 4:**
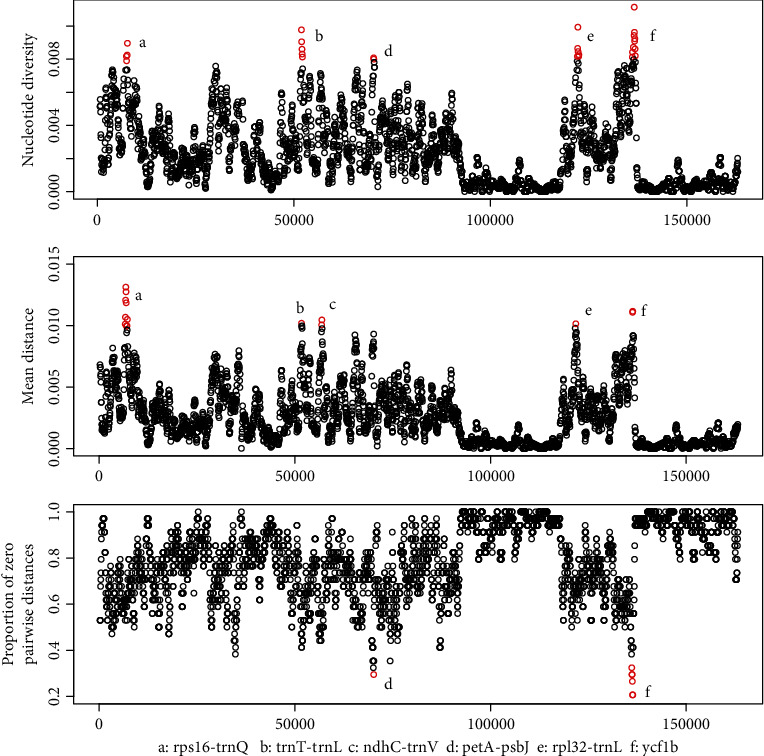
Specific DNA barcode development. Window length: 600 bp; step size: 50 bp; *x*-axis: position of the midpoint of a window. The three figures are the nucleotide diversity (*π*), mean sequence distance (*D*), and the proportion of zero pairwise distances (*Z*) among the species.

Using the slide window method, *π* values ranged from 0 to 0.01113 in a 600 bp window size, the *D* values ranged from 0 to 0.0202, and *Z* values ranged from 0.2059 to 1. We considered variable regions with *π* values > 0.008, *D* values > 0.01, and *Z* values < 0.3. Six variable regions (*rps16-trnQ*, *trnT-trnL*, *ndhC-trnV*, *petA-psbJ*, *rpl32-trnL*, and *ycf1b*) were identified in the *Styrax* chloroplast genome (Figure 4). These regions included five intergenic regions (*rps16-trnQ*, *trnT-trnL*, *ndhC-trnV*, *petA-psbJ*, and *rpl32-trnL*), and one was the coding region of *ycf1* (*ycf1b*). Four intergenic regions (*rps16-trnQ*, *trnT-trnL*, *ndhC-trnV*, and *petA-psbJ*) were located in the LSC region, and *rpl32-trnL* and *ycf1b* were located in the SSC region.

The percentage of variable sites among these six regions ranged from 4.36 to 5.85, and the parsimony-informative sites ranged from 1.61 to 3.85. According to the *π* values, *ycf1b* showed the highest variability in *Styrax*, followed by *petA-psbJ*, *rps16-trnQ*, *rpl32-trnL*, *trnT-trnL*, and *ndhC-trnV*. Using the distance methods, *ycf1b* had 92.56% discriminatory power, followed by *trnT-trnL* and *petA-psbJ*. Combined with *ycf1b* and *trnT-trnL*, all *Styrax* species were successfully distinguished. The tree-based results are presented in Figure 3(b). Compared to universal DNA barcodes, the combination of *ycf1b* and *trnT-trnL* had a higher resolution. Thus, *ycf1b* and *trnT-trnL* were chosen as *Styrax*-specific chloroplast DNA barcodes. The primers designed for the two regions are listed in Table S4, and the primers were tested to work well.

### 3.5. Superbarcode of *Styrax*

**Figure 5:**
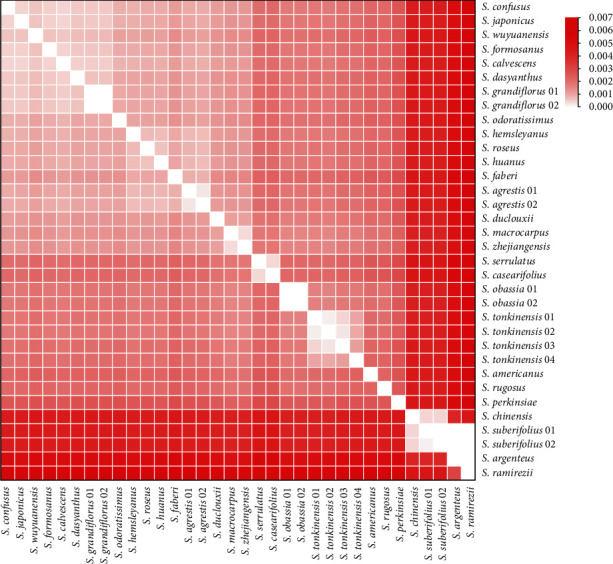
Pairwise genetic distances among *Styrax* samples.

**Figure 6:**
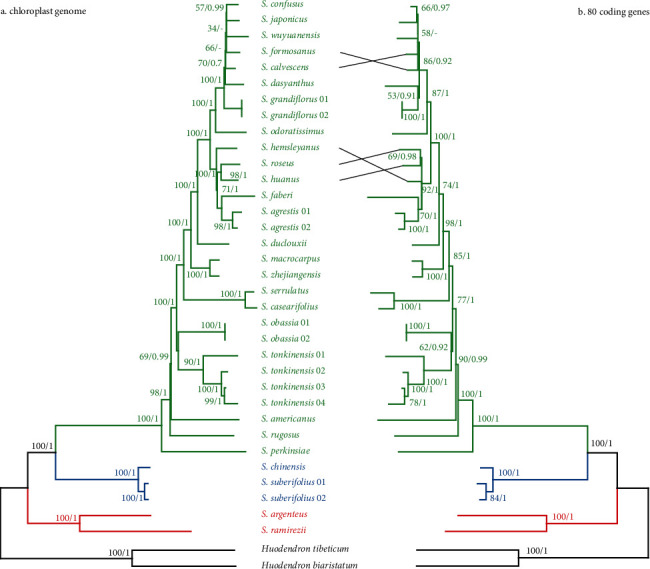
Phylogenetic trees of *Styrax*: (a) ML tree based on the whole chloroplast genome; (b) ML tree using 80 coding genes. ML bootstrap support value/Bayesian posterior probability presented at each node.

The 47 whole *Styrax* chloroplast genomes had an aligned length of 163,099 sites with 3,160 variable sites (1.94%) and 1,481 parsimony-informative sites (0.91%). The mean nucleotide diversity was found to be 0.00231 (Table 4). The genetic *P* distance of *the Styrax* species is shown in Figure 5. The mean genetic distance was 0.00244, the lowest divergence (0.0003) was between *S. macrocarpus* and *S. zhejiangensis*, and the largest sequence divergence (0.00611) was between *S. casearifolia* and *S. ramirezii*. The discriminatory power of the whole chloroplast genome as a DNA barcode was assessed using distance- and tree-based methods. Compared to the universal DNA barcodes or the six newly specific DNA barcodes, the whole chloroplast genome data exhibited the highest discriminatory power (Table 4 and Figure 6).

### 3.6. Phylogenetic Inference

The phylogenetic tree inferred from the chloroplast genome and 80 coding gene datasets was similar to the phylogenetic relationships of *Styrax* species (Figure 6). The best-fit model GTR+G from ModelFinder was used for the ML and BI analyses. The topologies of the ML and BI trees and the two datasets were nearly identical. All *Styrax* species formed a monophyletic clade (BS = 100/PP = 1), and some notes had shortened branches, indicating low divergence among some *Styrax* species. Three lineages were formed in the phylogenetic tree. *Styrax ramirezii* and *S. argenteus* were the first diverging branches (series *Valvatae*) and were sisters to the remaining species. *Styrax chinensis* and *S. suberifolius* formed the second lineage (series *Benzoin*). The remaining species formed the third lineage (series *Cyrta*) with a 100% bootstrap value.

## 4. Discussion

### 4.1. *Styrax* Chloroplast Genome Evolution

The Styrax chloroplast genomes were similar to other angiosperms, indicating that the chloroplast genome was a quadripartite structure, including a large single-copy, a small single-copy, and a pair of inverted repeats. The *Styrax* chloroplast genomes have highly similar genome structures, genome sizes, and gene contents (Figure 1), and the single-copy regions and noncoding regions are more variable than the IRs and coding regions (Figure S1).

SSRs, which consist of tandemly repeated motifs of six base pairs (bp) or less, are important markers for population genetics and germplasm management [5, 40, 41]. In the chloroplast genome, SSRs are dominated by mono- and dinucleotide repeats and A/T bases are the most common [7, 42, 43]. This was consistent with previous findings that the chloroplast genome is usually composed of polyA and polyT repeats [44]. A total of 61–74 SSRs were found in the *Styrax* chloroplast genomes (Figure 2), which were more abundant than other species of Styracaceae [45].

Small inversions have been found in most related species [46–48]. All of the inversions formed stem-loop structures, and there was no correlation between the lengths of inversions and inverted repeats [7]. Many small inversions are generated by parallel or back mutation events during chloroplast genome evolution [49, 50]. The inversion in *trnS^GCU^–trnG^GCC^* and *petN–psbM* occurred in some samples of *S. tonkinensis*, and *trnS^GCU^–trnG^GCC^* and *psaJ–rpl33* occurred in only one sample of *S. agrestis*. These inversions did not show phylogenetic signals (Table S3). Recent studies suggest that some small inversions are valuable for phylogenetic relationships in some groups [51–54].

### 4.2. Species Identification from Genes to Genomes

Rapid and accurate species delimitation is very important in biology. Morphological characteristics are the key methods used to identify the samples [55]. DNA barcoding is a new effective tool widely used in species identification since 2003 [56]. Selecting a DNA marker as a universal DNA barcode is essential for the diversity of organisms [57]. However, the selection of universal barcode(s) in plants is more complex than other organisms. The CBOL Working Group recommended three chloroplast markers (*rbcL*, *matK*, and *trnH-psbA*) and nuclear ITS as universal DNA barcodes for higher plants [58]. More evidence has shown that these markers have lower variability and discrimination power [15, 59–61]. This study assessed the three chloroplast markers in *Styrax* to evaluate their suitability for species resolution. Using the distance- and tree-based methods, their discrimination power was barely satisfactory (Table 3). Combining the two core DNA barcodes (*rbcL* and *matK*) had a resolution rate of less than 50%. ITS is regarded as a powerful phylogenetic marker at the species level, showing high interspecific divergence [62]. However, phylogenetic resolution using ITS data was also limited in *Styrax* [23].

The chloroplast genome sequence mutations (SNPs and indels) were not random and clustered into mutation hotspot regions, and these regions were selected as specific DNA barcodes [5, 59, 60, 63]. Specific DNA barcodes revealed a higher resolution rate than universal DNA barcodes. For example, *Oryza* chloroplast genomes were compared and five or six specific DNA barcodes for *Oryza* were identified [14, 64]. Using comparison of oak species, two intergenic regions *matK-trnK-rps16* and *trnR-atpA* and two coding regions *ndhF* and *ycf1b* were developed as specific DNA barcodes [15].

This study identified six variable markers (*rps16-trnQ*, *trnT-trnL*, *ndhC-trnV*, *petA-psbJ*, *rpl32-trnL*, and *ycf1b*). These markers had higher variable and species resolutions than the universal DNA barcodes (Table 3). According to the success discrimination rate, two markers (*trnT-trnL* and *ycf1*b) were selected as *Styrax*-specific chloroplast DNA barcodes. *TrnT-trnL* is an intergenic spacer region and has been frequently used in plant phylogeny [65, 66]. Dong et al. were the first to report *ycf1a* and *ycf1b* markers, located in the second-longest gene *ycf1* [60, 61]. *ycf1b* was more variable than the two core DNA barcodes, *rbcL* and *matK* [61]. Combining *ycf1*b and *trnT-trnL* significantly improved the identification success rate, and these two markers were chosen as the *Styrax*-specific DNA barcodes.

The advent of next-generation sequencing (NGS) technologies has led to a decrease in the cost of genome sequencing. Genomic data have extended the concept of the DNA barcoding approach, referred to as “superbarcoding” [14, 16], “ultrabarcoding” [67], or “plant barcoding 2.0” [17]. Compared to the nuclear and mitochondrial genomes, the chloroplast genomes were easily sequenced using genomic skimming [68–70], and the chloroplast genome has sufficient sequence variation in closely related species [6]. More studies showed that the chloroplast genome had a sufficiently high mutation rate which enables species identification and it may be best suited as superbarcodes for plants [70]. For example, using the chloroplast genome sequences, all 20 sampled *Olea* species had been successfully distinguished and even some subspecies of *O. europaea* can be identified [70]. Wu et al. also indicated that the chloroplast genome can be used to effectively differentiate *Fritillaria* species [71]. Moreover, chloroplast genome data have been widely used in plant phylogenetics at different taxonomic levels [8, 72, 73]. In this study, the chloroplast genome showed sufficient information for *Styrax* species identification (Table 4 and Figure 6). In addition to assembling the chloroplast genomes, clean reads from NGS could further be used to retrieve nuclear genome sequences, giving the possibilities for accurate species identification and phylogenetic relationship reconstruction.

## 5. Conclusions

The analyzed 34 *Styrax* chloroplast genomes have a similar structure, gene number, and gene order. SSR sites and small inversions were also identified. Comparisons of the *Styrax* chloroplast genome sequence divergences revealed that *rps16-trnQ*, *trnT-trnL, ndhC-trnV*, *petA-psbJ*, *rpl32-trnL*, and *ycf1b* were variable markers. Furthermore, *ycf1b* and *trnT-trnL* were suggested as *Styrax*-specific DNA barcodes. The whole chloroplast genome is potentially available as a superbarcode for *Styrax* species. This study demonstrated the potential of chloroplast genome data to improve the phylogenetic resolution.

## Figures and Tables

**Table 1:** Summary statistics for the assembly of 17 *Styrax* chloroplast genomes.

	LSC	IR	SSC	Total	GC%	Number of genes	Protein-coding genes	tRNA	rRNA
*Styrax agrestis*	87,495	26,048	18,279	157,870	37.0	114	80	30	4
*Styrax americanus*	87,448	26,053	18,272	157,826	37.0	114	80	30	4
*Styrax argenteus*	87,898	26,017	18,343	158,275	36.9	114	80	30	4
*Styrax casearifolius*	87,526	26,048	18,310	157,932	37.0	114	80	30	4
*Styrax formosanus*	87,664	26,047	18,295	158,053	36.9	114	80	30	4
*Styrax hemsleyanus*	87,586	26,048	18,293	157,975	36.9	114	80	30	4
*Styrax huanus*	87,507	26,060	18,290	157,917	37.0	114	80	30	4
*Styrax japonicus*	87,250	26,047	18,297	157,641	37.0	114	80	30	4
*Styrax obassia*	87,528	26,051	18,279	157,909	37.0	114	80	30	4
*Styrax perkinsiae*	87,444	26,047	18,272	157,810	37.0	114	80	30	4
*Styrax roseus*	87,546	26,034	18,292	157,906	37.0	114	80	30	4
*Styrax rugosus*	87,755	26,041	18,412	158,249	37.0	114	80	30	4
*Styrax serrulatus*	87,489	26,049	18,313	157,900	37.0	114	80	30	4
*Styrax suberifolius*	88,656	26,342	17,993	159,333	36.9	114	80	30	4
*Styrax tonkinensis*	87,642	26,049	18,318,	158,058	36.9	114	80	30	4
*Styrax tonkinensis*	87,553	26,049	18,317	157,968	36.9	114	80	30	4
*Styrax tonkinensis*	87,622	26,352	18,027	158,353	36.9	114	80	30	4

**Table 2:** The size and locations of small inversions in the *Styrax* chloroplast genomes.

Region	Position	Location	Length (bp)	
			Loop	Stem
LSC	*trnS^GCU^–trnG^GCC^*	Spacer	4	12
LSC	*atpF–atpH*	Spacer	3	14
LSC	*rpoC1*	Intron	4	10
LSC	*trnC^GCA^–petN*	Spacer	4	8
LSC	*petN–psbM*	Spacer	10	17
LSC	*trnS^UGA^–psbZ*	Spacer	164	11
LSC	*ycf3–trnS^GGA^* 01	Spacer	4	12
LSC	*ycf3–trnS^GGA^* 02	Spacer	4	7
LSC	*trnT^UGU^–trnL^UAA^*	Spacer	5	14
LSC	*trnF^GAA^–ndhJ* 01	Spacer	6	6
LSC	*trnF^GAA^–ndhJ* 02	Spacer	3	9
LSC	*trnF^GAA^–ndhJ* 03	Spacer	10	15
LSC	*petA–psbJ*	Spacer	27	15
LSC	*psaJ–rpl33*	Spacer	9	28
LSC	*clpP*	Intron	13	14
IR	*trnI^GAU^*	Intron	16	18
IR	*trnR^ACG^–trnN^GUU^*	Spacer	8	11

**Table 3:** The variability of the three universal DNA barcodes and six variable markers in *Styrax*.

Markers	Length	Variable sites		Parsimony-informative sites		Discrimination success (%) based on distance method	Nucleotide diversity
		Numbers	%	Numbers	%		
*rbcL*	695	14	2.01	9	1.29	18.52	0.00297
*matK*	878	25	2.85	13	1.48	44.44	0.00322
*trnH-psbA*	486	25	5.14	14	2.88	48.15	0.00888
*rbcL+matK*	1,573	39	2.48	22	1.40	48.15	0.00311
*rbcL+matK+trnH-psbA*	2,059	64	3.11	36	1.75	70.37	0.00412
*rps16-trnQ*	1,173	63	5.37	34	2.90	51.85	0.00800
*trnT-trnL*	1,055	46	4.36	17	1.61	70.37	0.00761
*ndhC-trnV*	701	54	7.70	27	3.85	59.26	0.00544
*petA-psbJ*	718	42	5.85	17	2.37	70.37	0.00807
*rpl32-trnL*	1,296	69	5.32	34	2.62	59.26	0.00766
*ycf1b*	1,350	73	5.41	42	3.11	92.59	0.00816
*petA-psbJ+ycf1b*	2,068	115	5.56	59	2.85	92.59	0.00813
*trnT-trnL+ycf1b*	2,405	119	4.95	59	2.45	100.00	0.00795
Six-marker combination	6,293	347	5.51	171	2.72	100.00	0.00760

**Table 4:** Chloroplast genome sequence variable in the *Styrax*.

Regions	Length	Variable sites		Information sites		Nucleotide diversity
		Numbers	%	Numbers	%	
LSC	91,693	2,301	2.51	1,088	1.19	0.0031
SSC	19,066	649	3.40	302	1.58	0.00399
IR	26,172	106	0.41	46	0.18	0.00045
Complete cp genome	163,099	3,160	1.94	1,481	0.91	0.00231

## Data Availability

The 17 *Styrax* chloroplast genomes are available in the GenBank database (accession numbers: MZ285733 to MZ285749).

## Supplementary Materials

Supplementary Materials Table S1: taxa included in this study with locality, voucher, and GenBank accession numbers. The bold font samples were sequenced in this study. Table S2: summary of the sequencing data for 17 *Styrax* samples. Table S3: the information of small inversions. Table S4: the primers used for amplification the specific *Styrax* DNA barcodes. Figure S1: visualization of genome alignment of the *Styrax* chloroplast genomes. The *x*-axis showed the coordinate between the chloroplast genomes.
