# Supplementary material for: Chloroplast Genome Evolution and Species Identification of Styrax (Styracaceae)
Source: Biomed Res Int. 2022 Feb 24;2022:5364094. doi: 10.1155/2022/5364094 (PMC8893999; doi:10.1155/2022/5364094)

Chloroplast genome evolution and species identification of *Styrax* (Styracaceae)

Yun Song[1,2]*, Wenjun Zhao[1], Jin Xu[1,2], MingFu Li[1,2*], Yongjiang Zhang[1]

[1]Institute of Plant Inspection and Quarantine, Chinese Academy of Inspection and Quarantine (CAIQ), Beijing 100176, China,

[2]CAIQ center for Biosafety, Sanya 572025, China,

* Corresponding author

E-mail: sydef1016@163.com, limf9@sina.com

Table S1. Taxa included in this study with locality, voucher, and GenBank accession numbers. The bold font samples were sequenced in this study.

Table S2. Summary of the sequencing data for 17 *Styrax* samples.

Table S3. The information of small inversions.

Table S4. The primers used for amplification the specific *Styrax* DNA barcodes.

Figure S1. Visualization of genome alignment of the *Styrax* chloroplast genomes. The x-axis showed the coordinate between the chloroplast genome.

Figure S1. Visualization of genome alignment of the *Styrax* chloroplast genomes. The x-axis showed the coordinate between the chloroplast genome.

Table S1. Taxa included in this study with locality, voucher, and GenBank accession numbers.

Taxon	Locality	Preservation	Accession number of plastome	Voucher
Styrax agrestis 01	Ledong, Hainan, China	Silicagel	MZ285735	SY850806
Styrax agrestis 02	Ledong, Hainan, China	N/A	MT644192	NF2020090
Styrax americanus	Kankakee County,Illinois, USA	Herbarium	MZ285749	PE01618866
Styrax argenteus	Guanacaste Liberia Area de Conversacion Guanacaste,Costa Rica	Herbarium	MZ285745	PE02110301
Styrax calvescens	Huangshan, Anhui, China	N/A	MN560141	NF2019468
Styrax casearifolius	Jianghong, Yunnan, China	Silicagel	MZ285737	SY850801
Styrax chinensis	Nanning, Guangxi, China	N/A	MT648752	NF2020091
Styrax confusus	Huangshan, in Anhui, China	N/A	MN560142	NF2019903
Styrax dasyanthus	Luhe District, Nanjing, China	N/A	MN329776	NF2018097
Styrax duclouxii	Nanjing, Jiangsu, China	N/A	MN882545	NF2019128
Styrax faberi	Qiyun, Anhui, China	N/A	MN335255	NF2018669
Styrax formosanus	Nantou, Taiwan, China	Herbarium	MZ285742	PE01883300
Styrax grandiflorus 01	N/A	N/A	KX111381	F365
Styrax grandiflorus 02	N/A	N/A	MF179493	14CS8610
Styrax hemsleyanus	Lushi, Henan, China	Silicagel	MZ285748	SY850797
Styrax huanus	Nanchuan, Sichuan, China	Silicagel	MZ285741	SY850798
Styrax japonicus	Yuanqu, Shanxi, China	Silicagel	MZ285743	SY850800
Styrax macrocarpus	Yizhang, Hunan, China	N/A	MT435525	DG-2019-002

Styrax obassia 01	Jinzai, Anhui, China	Silicagel	MZ285733	SY850795
Styrax obassia 02	Nanjing, Jiangsu, China	N/A	MN560143	NF2019061
Styrax odoratissimus	Huangshan, Anhui, China	N/A	MN368610	NF2018038
Styrax perkinsiae	Fugong, Yunnan, China	Silicagel	MZ285734	SY850793
Styrax ramirezii	N/A	N/A	MG719843	N/A
Styrax roseus	Leibo, Sichuan, China	Silicagel	MZ285736	SY850792
Styrax rugosus	Jingdong, Yuannan, China	Silicagel	MZ285746	SY850791
Styrax serrulatus	Kachin State Putao District, Myanmar	Herbarium	MZ285738	PE02037081
Styrax suberifolius 01	Jianggangshan, Jiangxi, China	Silicagel	MZ285747	SY850789
Styrax suberifolius 02	N/A	N/A	MG719828	N/A
Styrax tonkinensis 01	Luhe, Nanjing, Jiangsu	N/A	MT075718	NF2019868
Styrax tonkinensis 02	Nanyue, Hunan, China	Silicagel	MZ285740	SY850788
Styrax tonkinensis 03	Suining, Hunan, China	Silicagel	MZ285739	SY850799
Styrax tonkinensis 04	Xinning, Hunan, China	Silicagel	MZ285744	SY850803
Styrax wuyuanensis	Wuyuan, Jiangxi, China	N/A	MW166213	JXAU 2020026
Styrax zhejiangensis	N/A	N/A	MG702338	N/A

Table S2. Summary of the sequencing data for 17 *Styrax* samples.

Species	Clean data no.	Mapped reads no.	Mapped to reference genome (%)	Chloroplast genome coverage (X)
Styrax agrestis 01	25487458	316904	1.24	301.10597
Styrax americanus	12864020	67059	0.52	63.733795
Styrax argenteus	24107816	626810	2.60	594.03886
Styrax casearifolius	32517270	258037	0.79	245.07731
Styrax formosanus	20748168	504977	2.43	479.24778
Styrax hemsleyanus	23759700	85455	0.36	81.141003
Styrax huanus	19975582	460290	2.30	437.21385
Styrax japonicus	11971102	718402	6.00	683.58041
Styrax obassia 01	23055492	984696	4.27	935.3767
Styrax perkinsiae	24176372	450934	1.87	428.61732
Styrax roseus	23868728	1702907	7.13	1617.6463
Styrax rugosus	40957798	204338	0.50	193.68653
Styrax serrulatus	32674632	91391	0.28	86.818556
Styrax suberifolius 01	22828072	130990	0.57	123.3172
Styrax tonkinensis 02	27165452	100781	0.37	95.643055
Styrax tonkinensis 03	31683486	680905	2.15	646.55975
Styrax tonkinensis 04	32586122	92366	0.28	87.493764

Table S3. The information of small inversions.

	trnS^{GCU}-trnG^{GCC}	atpF -atpH	rpoC1	trnC^{GCA}-petN	petN-psbM	trnS^{UGA}-psbZ	ycf3-trnS^{GGA} 01	ycf3-trnS^{GGA} 02	trnT^{UGU}-trnL^{UAA}	trnF^{GAA} -ndhJ 01	trnF^{GAA}-ndhJ 02	trnF^{GAA}-ndhJ 03	petA-psbJ	psaJ-rpl33	clpP	trnI^{GAU}	trnR^{ACG}-trnN^{GUU}
S. agrestis 01	yes																
S. agrestis 02														yes			
S. americanus								yes				yes		yes			
S. argenteus												yes	yes	yes			
S. calvescens		yes						yes						yes			
S. casearifolius								yes						yes			
S. chinensis			yes			yes		yes				yes	yes	yes			
S. confusus		yes						yes						yes			
S. dasyanthus		yes												yes		yes	
S. duclouxii				yes				yes						yes			
S. faberi														yes			
S. formosanus		yes						yes						yes			
S. grandiflorus 01		yes												yes			
S. grandiflorus 02		yes												yes			
S. hemsleyanus														yes			
S. huanus														yes		yes	
S. japonicus		yes						yes						yes			
S. macrocarpus								yes						yes			
S. obassia 01					yes			yes		yes		yes		yes			

S. obassia 02				yes			yes		yes		yes		yes			
S. odoratissimus													yes	yes	yes	
S. perkinsiae						yes	yes				yes		yes			
S. ramirezii			yes				yes	yes			yes	yes	yes			yes
S. roseus													yes			
S. rugosus							yes				yes		yes			
S. serrulatus							yes						yes			
S. suberifolius 01			yes		yes		yes				yes	yes	yes			
S. suberifolius 02			yes		yes		yes				yes	yes	yes			
S. tonkinensis 02							yes				yes		yes			
S. tonkinensis 03				yes			yes				yes		yes			
S. tonkinensis 04				yes			yes				yes		yes			
S. tonkinensis 01	yes						yes				yes		yes			
S. wuyuanensis		yes					yes			yes			yes			
S. zhejiangensis							yes						yes			

Table S4. The primers used for amplification the specific *Styrax* DNA barcodes.

Markers	Forward primer	Primer sequence 5' to 3'	Reverse primer	Primer sequence 5' to 3'
trnT-trnL	trnT-trnL-f	TAGGAATTTAATAAACTATT	trnT-trnL-r	GCTTTAAAATTAGGATCTCATTG
ycf1b	ycf1b-f	TCTTTTTACTATCCAGTTTCGAA	ycf1b-r	TGGTTTGAAAAACCTCTTGTAAC

Figure S1. Visualization of genome alignment of the *Styrax* chloroplast genomes. The x-axis showed the coordinate between the chloroplast genome.

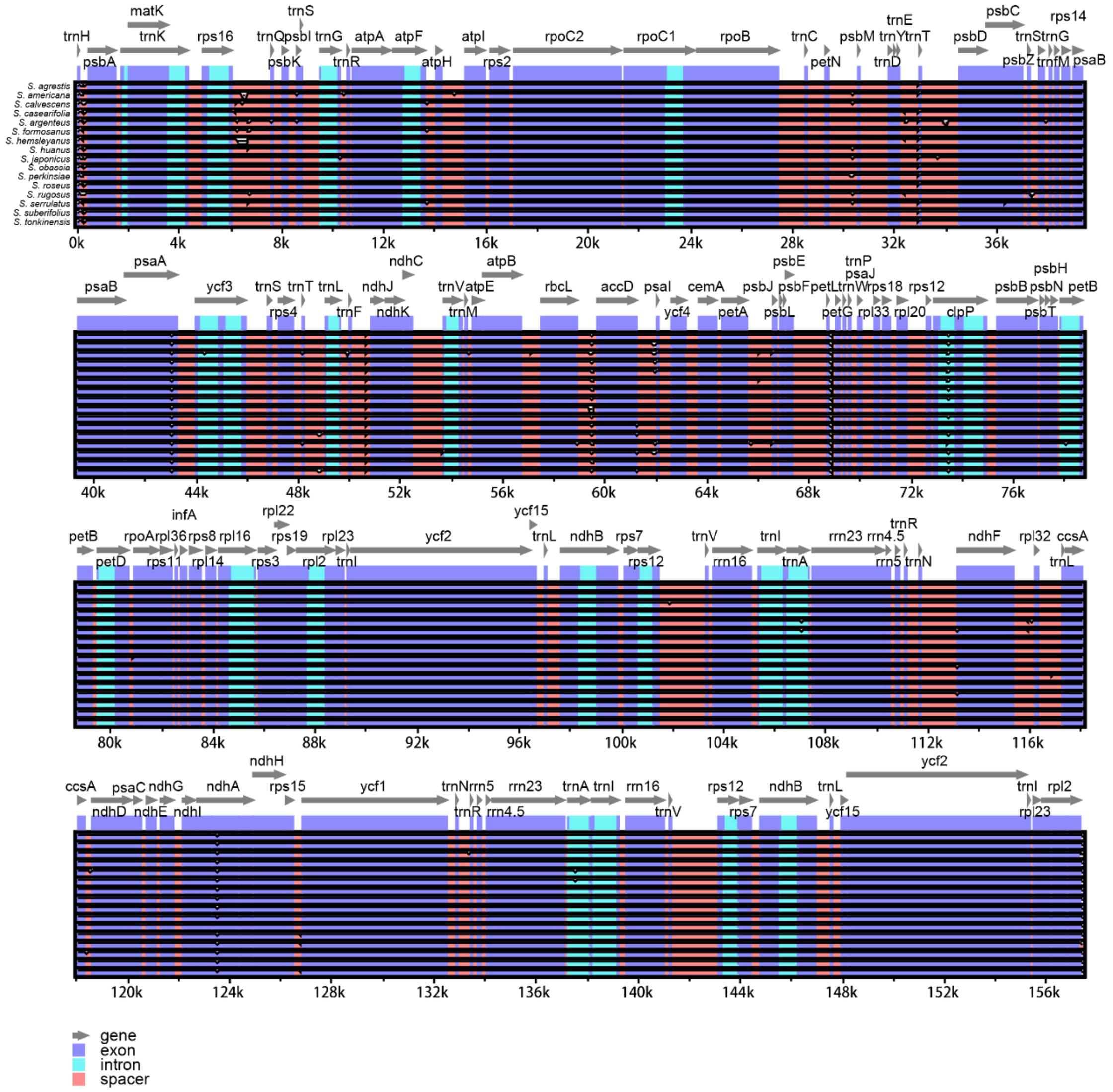

Supplement: Supplementary Materials — Table S1: taxa included in this study with locality, voucher, and GenBank accession numbers. The bold font samples were sequenced in this study. Table S2: summary of the sequencing data for 17 Styrax samples. Table S3: the information of small inversions. Table S4: the primers used for amplification the specific Styrax DNA barcodes. Figure S1: visualization of genome alignment of the Styrax chloroplast genomes. The x-axis showed the coordinate between the chloroplast genomes. [file 5364094.f1.pdf]
